# Dietary supplementation of phytoncide and soybean oil increases milk conjugated linoleic acid and depresses methane emissions in Holstein dairy cows

**DOI:** 10.1038/s41598-024-53799-2

**Published:** 2024-03-05

**Authors:** TaeBin Kim, MunHee Bae, JaeSung Lee, Jalil Ghassemi Nejad, HongGu Lee

**Affiliations:** Department of Animal Science and Technology, Sanghuh College of Life Sciences, Konkuk University, Seoul, 05029 Republic of Korea

**Keywords:** Phytoncide oil, Soybean oil, Conjugated linoleic acid, Methane, Systems biology, Zoology, Climate sciences, Environmental sciences

## Abstract

The objective of this study was to determine whether adding phytoncide oil (PO) and soybean oil (SBO) to the dairy cow diet could increase milk conjugated linoleic acid (CLA) and depress methane (CH_4_) emissions in Holstein dairy cows. Rumen fermentation was conducted at four levels of SBO (0, 1, 2, and 4%, on DM basis) and two levels of PO (0 and 0.1%, on DM basis) with in vitro experiment. To evaluate blood parameters, fecal microbe population, milk yield and fatty acid compositions, and CH_4_ production, in vivo experiment was conducted using 38 Holstein dairy cows divided into two groups of control (fed TMR) and treatment (fed TMR with 0.1% PO and 2% SBO as DM basis). In the in vitro study (Experiment 1), PO or SBO did not affect rumen pH. However, SBO tended to decrease ruminal ammonia-N (*p* = 0.099). Additionally, PO or SBO significantly decreased total gas production (*p* = 0.041 and *p* = 0.034, respectively). Both PO and SBO significantly decreased CH_4_ production (*p* < 0.05). In addition, PO significantly increased both CLA isomers (c9, t11 and t10, c12 CLA) (*p* < 0.001). Collectively, 0.1% PO and 2% SBO were selected resulting in most effectively improved CLA and decreased CH_4_ production. In the in vivo study (Experiment 2), 0.1% PO with 2% SBO (PSO) did not affect complete blood count. However, it decreased blood urea nitrogen and magnesium levels in blood (*p* = 0.021 and *p* = 0.01, respectively). PSO treatment decreased pathogenic microbes (*p* < 0.05). It increased milk yield (*p* = 0.017) but decreased percentage of milk fat (*p* = 0.013) and MUN level (*p* < 0.01). In addition, PSO treatment increased both the concentration of CLA and PUFA in milk fat (*p* < 0.01). Finally, it decreased CH_4_ emissions from dairy cows. These results provide compelling evidence that a diet supplemented with PSO can simultaneously increase CLA concentration and decrease CH_4_ production with no influence on the amount of milk fat (kg/day) in Holstein dairy cows.

## Introduction

In response to the growing interest in health and eco-friendly substances, recent research has focused on enhancing the quality of ruminant dairy, particularly through the use of plant extracts like conjugated linoleic acid (CLA). CLA, a functional natural product synthesized in the rumen, has demonstrated numerous health benefits in humans, acting as an effective anticancer agent and exhibiting antioxidant properties^1–3^. Furthermore, CLA has been associated with improvements in atherosclerosis, immune response, cholesterol reduction, and diabetes prevention^4–7^. According to previous research, although CLA isomers may vary greatly, cis-9, trans-11 CLA is the main isomer^7^, and its role is the greatest among all isomers. Interestingly, cis-9, trans-11 CLA levels in ruminant products are higher than those in other animal products. This is because the cis-9, trans-11 CLA can be produced by microbes in the rumen of ruminant. To elevate cis-9, trans-11 CLA levels in ruminant products, soybean and sunflower supplements rich in linoleic acid have traditionally been used^8,9^. Their extract in oil form is known to be the direct source that can increase CLA^10^ and inhibit CH_4_ production^11,12^. While these plant oils effectively increase CLA, they may also elevate trans-10, cis-12 CLA, negatively impacting milk fat synthesis^13–15^. Addressing this concern, phytoncide oil (PO) has emerged as a promising alternative. Derived from various plants, PO contains antimicrobial compounds such as β-carotene, regulating bacteria involved in fatty acid bio-hydrogenation in the rumen^16–19^.

Kim et al. demonstrated that feeding dairy cows with Phytoncide oil extracted from Pine Nut Cones (*Pinus* *koraiensis*) increased CLA in milk without affecting milk fat. Additionally, the use of flavonoids and essential oils has shown potential in stimulating CLA accumulation during bio-hydrogenation, while mitigating methane production^16,20^.

According to Oh et al.^21^, plant extracts can increase propionate and decrease acetate. Propionate can reduce CH_4_ production in rumen^21,22^. Biohydrogenation is involved in the metabolism to produce CLA from linoleic acid. It is related to the formulation of CH_4_ and volatile fatty acid (VFA) because of their competition with hydrogen ion^12,23^. Building on this knowledge, we hypothesized that combining soybean oil (SBO) with phytoncide oil from pine nut cone (PO) could increase CLA yield particularly cis-9, trans-11 CLA in milk fat^9^ and decrease CH_4_ production^21,22^, while maintaining or increasing milk fat, by increasing ruminal propionate proportion and thus increasing VFA in rumen^8,9,21^.

There is a lack of information as to whether phytocides can affect final ruminant production and reduce CH_4_ emissions. By investigating both in vitro and in vivo effects, our objective is to contribute valuable insights into the impact of phytoncide oil supplementation on ruminant production and methane emissions. Therefore, the objective of this study was to investigate in vitro and in vivo effects of phytoncide oil plus soybean oil on increasing milk CLA and depressing CH_4_ emissions in Holstein dairy cows.

## Materials and methods

### Experimental materials and procedures for in vitro experiment

The procedures involving animals in this study followed the ARRIVE guidelines. All cannulation procedures involving animals were performed according to the Animal Experimental Guidelines in Konkuk University and the ethical approval was obtained by the Animal Care and Use Committee (IACUC) of Konkuk University, Republic of Korea (Approval No.: KU18058).

The ruminal fluid was collected after filtering with two layers of cheese cloth through rumen cannula at 2 h before feeding. The collected ruminal fluid was kept in a 2 L thermos bottle that was filled with preheated CO_2_ gas and with oxygen (O_2_) completely removed, maintained at 39 °C, and then moved to the research lab. After removing feed particles by vacuum pump, the supernatant was used for the test.

The basal diet was dried in a drying oven (JS-CO2-AT100, Johnsam Co., Ltd. Bucheon, Korea) at 60 °C for 24 h. The forage and concentrate were then crushed with a 2 mm screen and used as a TMR at a ratio of 6:4. Then 0.2 g of TMR and 20 mL of anaerobic McDougall buffer^24^ were added to a 60 mL serum bottle containing 10 mL of ruminal fluid. After adding 30 mL of culture solution to the culture bottle, two levels of PO (0 and 0.1% on DM basis arranged by Phylus Co., Ltd. Seoul, Korea) were added according to each treatment and four levels of SBO (0, 1, 2, and 4% on DM basis arranged by Haepyo Co., Ltd) as a precursor of CLA in the rumen were added, respectively. Each ruminal sample was kept at the maximum anaerobic conditions by flushing with CO_2_ gas and capping with a rubber cap and an aluminum cap to prevent gas leakage as much as possible. The experiment was conducted with 8 treatments (three replicates per treatment) at time intervals of 0, 6, 12, 24, 48 h in a shaking incubator (JSP Corp, SI-900R; 100 rpm) at 39 °C.

### Analysis

After each incubation, total gas and CH_4_ were detected and the portion of ruminal sample was distributed for analysis of pH, VFA, ammonia-N, and fatty acid. Measurement of total gas was conducted using a 50 mL gas cylinder (Habdong Co., Anyang, Korea). CH_4_ was detected using a Gas Chromatography/TCD (HP 6890, Hewlett Packard Co., CA, USA) equipped with an HP-PLOT-A column (dimension: 30 m × 0.32 mm × 20.00 μm) according to Owen et al.^25^. Culture solution of 2 mL was collected for ammonia (1 mL) and VFA analyses (1 mL). All samples collected were kept frozen at − 20 °C until analyzed. Ammonia concentration was determined with the method of Fawcett and Scott^26^ using a spectrophotometer (Model 680, BIO-RAD, CA, USA). Culture solution of 1 mL was mixed with 0.1 mL 25% phosphoric acid. Then 0.2 mL pivalic acid solution (2%, w/v) was added as an internal standard. The mixed solution was centrifuged at 15,000×*g* for 15 min and the supernatant was used to determine the concentration and composition of VFA using a gas chromatograph (HP 6890, Agilent Technologies, Santa Clara, CA, USA). Analysis of rumen fluid for long-chain fatty acids measurement was performed as described by Choi et al.^27,28^. In brief, long-chain fatty acids were extracted using 20 mL Folch's solution of lyophilized 200 mL rumen fluid^29^. In order to remove the feed particles from the rumen fluid, centrifugation was carried out at low speed (2000×*g*, 4 °C) for 10 min. The supernatant was collected and centrifuged again at high speed (22,000×*g*, 4 °C) for 10 min to separate bacteria. The lower layer of the centrifuged rumen evaporated at 60 °C. Using N_2_ gas, NaOH and BF_3_ were added to methylate free fatty acids into FA methyl ester (FAME), and then diluted in hexane. For the fatty acid analysis, a 7890B GC system equipped with a 7890 series auto-sampler, 7690B series injector, and flame ionization detector (FID) was used (Agilent Technologies Inc., Santa Clara, CA, USA). We used a SP™-2560 fused silica capillary column (100 µm inner diameter × 0.25 mm inner diameter, 0.2 µm thick, Supelco Inc., Bellefonte, PA, USA), and the oven temperature from 70 to 225 °C, and then 100 °C for 5 min. The hydrogen (H_2_) flow rate to the detector was 55 mL/min, the oxygen (air) flow rate was 400 mL/min, and the helium (He) flow rate was set to 20 mL/min. Fatty acid peaks were detected using Supelco^®^ 37 Component FAME Mix (47885-U, Supelco Inc., Bellefonte, PA, USA). The detected fatty acid peaks were trans-11-octadecenoic methyl ester (46905-U; USA), cis-9, trans-11-CLA, trans-10, and cis-12 CLA (Matreya LLC, Pleasant Gap, PA, USA) were used and included in the analysis. The detectable amounts of SA (Stearic acid, C18:0), TVA (trans-11 vaccenic acid, C18:1), LA (Linoleic acid, C18:2), LnA (α-linoleic acid, C18:3), cis-9 trans-11 CLA and trans-10 cis-12 CLA were calculated as the ratios of separately analyzed fatty acids.

### Animals and design for in vivo experiment

A total of 38 Holstein dairy cows (milk fat: 5.0 ± 0.30%; milk yield: 37.1 ± 2.85 kg/day/head; days in milk: 99.4 ± 24.32; parity: 3.1 ± 0.68) were randomly assigned to two groups to analyze milk fatty acids, CH_4_ production, fecal microbe population, and blood parameters for 35 days. The cows were housed in a roofed shelter equipped with sunlight-roof and bedded with dry manure for comfort (4.5 m^2^ per cow). The sunlight-roof covered the barn and winch curtain helped ventilation. The cows were fed in trough and each pen was equipped with separate water throughs. The procedures involving animals in this study followed the ARRIVE guidelines. The ethical procedure was according to the Animal Experimental Guidelines in Konkuk University and approved by the Animal Care and Use Committee (IACUC) of Konkuk University, Republic of Korea (Approval No.: KU18058).

### Diets and treatments

Dairy cows were fed total mixed ration (76.8%), roughage (8.4%), and concentrated feed (14.8%). The TMR and concentrated feed were also ready-made by feed companies. The roughage was oats hay. These experimental diets were formulated to meet or exceed the NRC recommendations^30^. They were provided for ad libitum intake. Supplemental Table S1 shows ingredients and chemical compositions of diet and the experimental treatments. According to results of in vitro experiment, PSO (0.1% PO and 2% SBO on DM basis) was mixed with the vehicle and top dressed onto the TMR. The control group (Control) received the same amount of TMR as the experimental group (not containing PO or SBO).

Feed samples were analyzed for dry matter (DM), crude protein, ether extract, crude fiber, crude ash, neutral detergent fiber (NDF), and acid detergent fiber (ADF) according to AOAC^31^ procedures. DM content was determined by drying samples in a vacuum oven at 100 °C overnight. Ash content was determined by incineration at 550 °C overnight in a muffle furnace. Crude protein (N×6.25) was determined using the Kjeltec System (Kjeltec 2400, FOSS, Hillerød, Denmark). Crude fiber, NDF, and ADF contents of feed samples were analyzed using a Fibertec System (Fibertec 2010, FOSS, Hillerød, Denmark). Ether extract (EE) contents were determined using an ether extraction system (ANKOM XT15 Extractor, ANKOM Technology, NY, USA). Mineral contents of the feed were determined with inductively coupled plasma optical emission spectrometry (ICP-OES, Thermo, Waltham, MA, USA).

### Blood collection and analysis

For blood cell counting and metabolite analysis, blood samples were collected from each Holstein dairy cow via a jugular vein at 3 h before feeding and on experimental days 0, 21, and 35. Using EDTA tube (BD 367844 Vacutainer, Becton Dickinson, NJ, USA), whole blood was subjected to complete blood count (CBC) test using a VetScan HM2 Hematology System (Abaxis, Union City, CA, USA). In addition, the plasma portion of blood was obtained by using heparin tube (BD 367874 Vacutainer, Becton Dickinson, NJ, USA) and centrifuging at 3000 rpm for 15 min in order to determine blood metabolites using a Chemistry analyzer (Furuno CA-270, Nishinomiya, Japan). The metabolites were: albumin, glutamic-oxaloacetic transaminase, glutamic pyruvic transaminase, blood urea nitrogen, creatinine, triglycerides, cholesterol, glucose, calcium, magnesium, non-ester fatty acid.

### Fecal collection and analysis of microbes

Feces were collected on the same day and time as blood sampling occurred. They were obtained from each Holstein dairy cow from the rectum, kept in liquid nitrogen, transferred into ice box at − 4 °C, transported to laboratory, and maintained in a deep freezer (− 80 °C) until analysis. One gram of fecal sample was diluted with 10 mL of 0.85% NaCl solution. Then 1 mL of the mixture was subjected to ten-fold serial dilution (10^1^ to 10^11^). The dilution was vigorously shaken with a vortex mixer. Then 0.1 mL of the dilution was spread onto a agar plate. Enumeration of bacteria was performed on MRS agar plates for *Lactobacillus* spp. and LB agar (Luria–Bertani broth diluted with bacto agar) plates for *bacillus* spp. Plates were incubated in anaerobic condition at 37 °C for 24 h. Under the same condition, enumeration was done for pathogenic microbes *Salmonella* and *E. coli* on MacConkey agar plates. The number of colony-forming unit (CFU) was expressed as ${{\text{log}}}_{10}\frac{CFU}{g}$ feces.

### CH_4_ emission analysis

At 0, 7, 21, and 35 days, measurement of CH_4_ emission was conducted using Laser Methane mini-Green (LMm-G; Tokyo gas engineering solution, Tokyo, Japan). Between 10:00 and 11:00 hours when gas emissions were the most active during feed intake, CH_4_ emission levels from all dairy cows in each group were measured in triplicates. To increase the reliability of measurement, a fixed distance was used for each measurement and external environment (temperature, humidity, wind speed) was also measured. Finally, the total ppm value was calculated for each treatment by dividing the value of ppm-m (parts per million × meter) to the distance (meters) measured after derivation.

### Milk collection and analysis

During the experimental period, milk was collected twice a day (at 3 am and 3 pm). Total milk yield was then calculated, and the mean milk yield for 7 days was measured. At the end of the experiment, average milk yield was calculated. After mixing the collected milk in the morning and afternoon at each sampling day (0, 21 and 35 days), samples were stored at 4 °C for milk composition analysis. The subsamples were stored at − 20 °C for fatty acids analysis.

Milk composition was measured with a MilkoScan (CombiFoss FT + 500 S/H, Hillerød, Denmark). Milk lipids were extracted and detected using the same methods as described above for the in vitro experiment. Analysis conditions were: Sp-2560 capillary column (dimension: 100 m × 0.25 mm × 0.2 μm film thickness); injection:split, 30:1; heater temperature, 255 °C; pressure, 32.64 psi; total flow, 39.5 mL/min; split flow, 36.0 mL/min; injection volume, 1.0 μL; carrier gas, helium 1.2 mL/min; oven program, 70–100 °C at 5 °C/min and hold for 2 min, then 100–175 °C at 10 °C/min and hold for 40 min, then 175–225 °C at 5 °C/min and hold for 40 min; detector, FID System; and heater temperature, 260 °C (H_2_ flow: 40 mL/min, air flow: 400 mL/min).

### Statistical analyses

Results obtained from in vitro and in vivo experiments were subjected to least squares analysis of variance according to ANOVA and MIXED procedure of SAS (SAS Inst., Inc., Cary, NC, USA), respectively. Duncan multiple range test was used for ranking treatment means within a significant *F* test. The normality of the data distribution was assessed using the univariate procedure in SAS. This allowed us to verify whether the data met the assumption of normality, which is a critical aspect of conducting accurate statistical analyses. Animals in in vivo were randomly assigned to control and treatment groups based on completely randomized design. Statistical differences were considered significant at *P* < 0.05. Differences among means with 0.05 < *P* < 0.10 were accepted as representing tendencies of differences.

## Results

### In vitro experiment

The changes in ruminal pH, ammonia-N (NH_3_-N) and gas production are shown in Table 1. Oil supplementation materials did not affect the pH of the culture solution over the incubation time. No significant differences were observed in ammonia-N concentration by phytoncide oil. However, numerically lower values were observed in phytoncide oil treatments. In contrast, ammonia concentration tended to increase by SO treatment (*p* = 0.099).

**Table 1:** Effects of phytoncide essential oil and soybean oil on ruminal pH, NH_3_-N and gas production.

Item	Incubation time (h)	Treatment^1^								SEM	P-value		
		PO, 0%				PO, 0.1%					PO	SO	PO × SO
		SO				SO							
		0%	1%	2%	4%	0%	1%	2%	4%				
pH	0	6.29	6.30	6.31	6.29	6.28	6.29	6.30	6.30	0.007	0.225	0.435	0.526
	6	6.27	6.29	6.28	6.28	6.25	6.26	6.25	6.27	0.015	0.315	0.288	0.321
	12	6.21	6.20	6.21	6.20	6.19	6.19	6.20	6.19	0.008	0.202	0.273	0.626
	24	6.18	6.17	6.17	6.17	6.18	6.18	6.16	6.17	0.004	0.236	0.199	0.335
	48	6.11	6.12	6.10	6.13	6.12	6.11	6.09	6.10	0.002	0.261	0.325	0.521
NH_3_-N (mg/100 mL)	0	12.17	12.14	12.15	12.14	12.25	12.25	12.25	12.25	0.092	0.522	0.211	0.582
	6	12.75	12.61	12.68	12.645	15.74	14.31	15.02	14.66	1.037	0.422	0.182	0.656
	12	16.43	16.07	16.25	16.16	18.39	18.38	18.38	18.38	1.161	0.164	0.144	0.908
	24	20.12	19.53	19.82	19.67	21.04	22.45	21.74	22.09	1.319	0.215	0.101	0.377
	48	29.94	29.78	29.86	29.82	23.78	28.53	26.15	27.34	0.753	0.156	0.099*	0.428
Total gas (mL)	6	14.31	12.96	10.62	9.45	14.56	11.88	10.31	8.24	0.225	0.136	0.045**	0.088
	12	20.03	16.07	15.71	14.58	19.44	15.47	14.77	11.82	0.642	0.115	0.033**	0.095
	24	24.85	19.89	20.88	21.51	25.32	18.05	19.24	15.39	0.851	0.215	0.053**	0.102
	48	30.50	24.12	25.02	24.75	29.76	23.27	22.62	18.09	0.461	0.041**	0.034**	0.072
Methane gas (mL)	6	1.41	1.19	1.00	0.89	0.98	0.89	0.74	0.60	0.034	0.014**	0.01**	0.044**
	12	2.43	1.55	1.65	1.64	1.65	1.53	1.03	0.95	0.488	0.002**	0.005**	0.062
	24	3.19	2.12	2.45	2.51	2.65	1.95	1.63	1.45	0.515	< 0.001**	0.003**	0.012**
	48	4.61	2.57	2.87	3.01	3.42	2.49	1.76	1.95	0.563	< 0.001**	0.005**	0.003**

*P < 0.1, **P < 0.05.

^1^Treatments: *PO* phytoncide oil, *SO* soybean oil, mg.

*SEM* standard error of the mean.

The production of total gas and CH_4_ was detected by using GC at 0, 12, 24 and 48 h incubation time. There were significant decreases in the production of total gas and CH_4_ among treatment groups (*p* < 0.05). Total gas production was lower (*p* = 0.041 and 0.034, respectively) when PO or SBO was added, especially from 48 h incubation. Additionally, from 6 h incubation, CH_4_ production was lower (*p* < 0.05) in treatment groups containing PO or soybean oil (SBO). Except for 12 h incubation, CH_4_ was much lower (*p* < 0.05) when both PO and SBO were added to the diet, compared with separate supplementation and the most decreases in CH_4_ was observed when 0.1% PO and 2% SBO supplementation.

The properties of volatile fatty acids at 48 h incubation time were analyzed by using GC (Table 2). There was no significant difference in individual VFAs.

**Table 2:** Effects of phytoncide essential oil and soybean oil on volatile fatty acid (VFA) in rumen fluid.

Item	Treatment^1^								SEM	P-value		
	PO, 0%				PO, 0.1%					PO	SO	PO × SO
	SO				SO							
	0%	1%	2%	4%	0%	1%	2%	4%				
Individual VFAs (mM) at 48 h												
Acetic acid (C2)	90.40	83.82	85.23	86.75	78.25	78.42	77.82	78.43	3.611	0.716	0.265	0.451
Propionic acid (C3)	41.31	38.92	40.12	40.39	39.72	36.85	35.22	37.53	2.643	0.096*	0.243	0.398
Iso-butyric acid (C4)	0.45	0.43	0.43	0.55	0.32	0.36	0.37	0.46	0.551	0.671	0.36	0.256
Butyric acid (C4)	19.59	18.91	20.65	19.99	17.77	18.72	20.37	19.22	1.422	0.491	0.082*	0.682
Iso-valeric acid (C5)	3.73	3.58	3.70	3.94	3.43	3.65	3.67	3.85	0.242	0.888	0.382	0.399
Valeric acid (C5)	2.68	2.54	2.67	2.90	2.66	3.00	2.84	3.10	0.201	0.168	0.486	0.174
A:P (C2/C3)	2.19	2.15	2.12	2.15	1.97	2.13	2.21	2.09	1.37	0.132	0.344	0.281

*P < 0.1.

^1^Treatments: *PO* phytoncide oil, *SO* soybean oil, mg.

*SEM* standard error of the mean.

In the variation of long chain fatty acids. From 6 to 48 h, the trans vaccenic acid (TVA) was higher (*p* < 0.05) in treatment groups containing PO or SBO, except for 4% dosage (Table 3). Additionally, linoleic acid was higher (*p* < 0.001) at all times in the treatment groups containing PO or SBO. From 24 h incubation, both conjugated linoleic acids (c9, t11 CLA, t10, c12 CLA) were higher (*p* < 0.001, *p* < 0.05), and total CLA was significantly higher (*p* < 0.001) in the PO treatment groups than the non-PO treatment groups. Additionally, soybean oil significantly increased CLA at 24 and 48 h after incubation (*p* < 0.05 at 24 h, *p* < 0.001 at 48 h). Moreover, higher CLA level from 24 to 48 h was observed in the treatment group containing more SBO with PO (*p* < 0.05).

**Table 3:** Effects of phytoncide essential oil and soybean oil on long chain fatty acids (LCFAs) in rumen fluid.

LCFA (mg/mL)	Treatment^1^								SEM	P-value		
	PO, 0%				PO, 0.1%					PO	SO	PO × SO
	SO				SO							
	0%	1%	2%	4%	0%	1%	2%	4%				
0 h												
SA	0.701	0.691	0.731	0.798	0.687	0.697	0.672	0.779	0.021	0.783	0.462	0.911
TVA	0.041	0.044	0.048	0.047	0.036	0.032	0.037	0.042	0.003	0.719	0.910	0.417
LA	0.035	2.061	4.121	7.552	0.036	2.091	4.182	7.572	0.760	< 0.001**	< 0.001**	0.891
LnA	0.009	0.011	0.013	0.016	0.007	0.008	0.010	0.012	0.002	0.781	0.522	0.150
6 h												
SA	0.771	0.760	0.804	0.878	0.756	0.767	0.739	0.857	0.610	0.031	0.512	0.124
TVA	0.039	0.041	0.061	0.098	0.051	0.056	0.091	0.081	0.009	< 0.001**	< 0.001**	0.212
LA	0.021	1.041	2.081	4.111	0.024	1.076	2.151	4.231	0.381	< 0.001**	0.032**	0.412
LnA	0.006	0.007	0.009	0.010	0.007	0.008	0.010	0.012	0.001	0.790	0.736	0.124
12 h												
SA	0.848	0.836	0.885	0.966	0.831	0.843	0.813	0.943	0.033	0.230	0.731	0.699
TVA	0.042	0.045	0.057	0.075	0.024	0.046	0.066	0.064	0.008	0.080	0.373	0.518
LA	0.012	0.581	1.162	2.116	0.011	0.665	1.329	2.226	0.038	< 0.001**	< 0.001**	0.525
LnA	0.009	0.011	0.013	0.016	0.007	0.008	0.010	0.012	0.001	0.210	0.125	0.325
24 h												
SA	1.601	1.621	1.712	1.801	1.555	1.626	1.623	1.799	0.126	0.337	0.162	0.893
TVA	0.050	0.053	0.068	0.089	0.029	0.055	0.079	0.076	0.053	0.091*	0.119	0.912
LA	0.010	0.465	0.930	1.693	0.009	0.532	1.063	1.781	0.174	< 0.001	< 0.001	0.411
LnA	0.011	0.013	0.016	0.019	0.008	0.010	0.012	0.015	0.022	0.426	0.621	0.776
Sum of CLAs	ND	0.109	0.161	0.294	ND	0.123	0.269	0.452	0.189	< 0.001	0.047**	0.031**
c9,t11 CLA	ND	0.088	0.131	0.242	ND	0.114	0.224	0.381	0.141	< 0.001	0.051*	0.128
t10,c12 CLA	ND	0.021	0.030	0.052	ND	0.009	0.045	0.071	0.078	0.031**	< 0.001**	0.624
48 h												
SA	1.913	1.837	1.996	2.152	1.894	1.921	1.874	2.152	0.127	0.141	0.102	0.451
TVA	0.055	0.059	0.062	0.072	0.055	0.046	0.051	0.072	0.011	0.112	0.049**	0.902
LA	0.009	0.301	0.672	1.092	0.007	0.281	0.728	1.210	0.428	< 0.001**	< 0.001**	0.268
LnA	0.008	0.009	0.011	0.013	0.006	0.007	0.008	0.010	0.002	0.290	0.217	0.933
Sum of CLAs	ND	0.149	0.298	0.522	ND	0.232	0.472	0.710	0.115	< 0.001**	< 0.001**	0.045**
c9,t11 CLA	ND	0.121	0.241	0.444	ND	0.201	0.401	0.622	0.108	< 0.001**	< 0.001**	0.032**
t10,c12 CLA	ND	0.029	0.057	0.078	ND	0.031	0.071	0.088	0.048	0.021**	< 0.001**	0.082*

*P < 0.1, **P < 0.05.

^1^Treatments: *PO* phytoncide oil, *SO* soybean oil, mg.

*SEM* standard error of the mean.

*LA linoleic acid (C18:2), *LnA* linolenic acid (C18:3), *TVA* trans-11 vaccenic acid (C18:1), *SA* stearic acid (C18:0), *LCFAs* long chain fatty acids, *ND* not detectable.

Collectively, 0.1% PO and 2% SBO supplementation on TMR was selected for in vivo study because its dosage indicated most activation of inducing hydrogen ion from CH_4_ production to biohydrogenation of fatty acids when considering the result of most decrease in CH_4_ and increase in CLA simultaneously with no influence on ruminal fermentation.

### In vivo experiment

#### Blood analysis

The result of CBC analysis was conducted by using whole blood (Supplemental Table S2). There were no significant differences in white blood cells, red blood cells, hemoglobin, hematocrit, mean corpuscular volume, and mean corpuscular hemoglobin or mean corpuscular hemoglobin concentration between the PSO treatment and control groups. In the result of the blood metabolic parameters (Supplemental Table S3), There was no significant difference (*p* > 0.05) in most of the parameters, except for blood urea nitrogen (BUN) and magnesium (MG). The two metabolites, BUN and MG, were lower (*p* = 0.021, *p* = 0.01) in the PSO treatment group.

#### Fecal microbes

**Figure 1:**
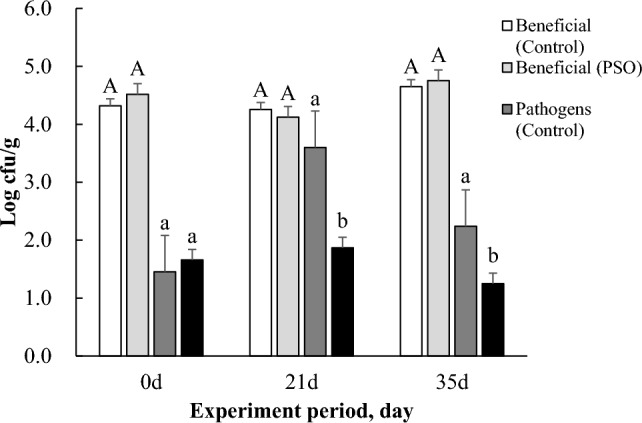
Effects of the mixture on fecal coliform and beneficial microbe population (35 days). Values are expressed as means. Contrasts of the same microbes between Control and PSO in each period are shown (e.g. Beneficial-Control vs Beneficial-PSO). *Differences in superscript indicate significance at *P* < 0.05. Beneficial fecal microbes: Bacillus and *L. bacillus* spp; Pathogenic fecal microbes: *E. coli* and *Salmonella* spp.

The beneficial microbes in the fecal were not affected by the treatment. However, the pathogenic microbes were significantly lower (*p* < 0.05) from 21 to 35 days in the fecal of the PSO treatment group (Fig. 1).

#### Milk yield, composition, and fatty acids

There was no difference in milk yield between the groups from the initiation of the study until 2 weeks. However, from 15 days, an increase (*p* < 0.01) in milk yield was observed in the PSO group (Table 4). Thus, the total average of milk yield was higher (*p* = 0.017) in the PSO group than in the control group. Although milk fat content (%) was lower in PSO group, 4% FCM was not difference between two groups. In addition, lower value of MUN was observed (*p* < 0.01) in the treatment group (Table 5). Although the metabolites of CLA, stearic acid, or trans vaccenic acid were not changed consistently, cis-9, trans-11 CLA in milk was higher at nearly double (*p* < 0.01), and trans-10, cis-12 CLA was higher (*p* < 0.05) in the treatment group. Additionally, the total CLA was higher (*p* < 0.01) in the PSO group (Table 6).

**Table 4:** Effect of feeding mixed phytoncide essential oil and soybean oil to dairy cow on milk yield.

Period (d)	Control	PSO^1^	SEM	P-value
	Milk yield, kg			
0	33.6	34.3	1.32	0.807
1–7 (AVG)	34.0	35.0	1.31	0.334
8–14 (AVG)	33.9	35.1	1.25	0.202
15–21 (AVG)	32.7	35.8	1.32	< 0.01
22–28 (AVG)	32.2	35.9	1.37	< 0.01
29–35 (AVG)	32.8	35.4	1.34	0.012
1–35 (AVG)	33.1	35.4	1.30	0.017

^1^PSO: group fed TMR with 0.1% PO and 2% SBO as DM basis.

**Table 5:** Effects of feeding mixed phytoncide essential oil and soybean oil to dairy cows for 35 days on milk compositions.

Items^2^ (%)	Control			SEM	PSO^1^			SEM	P-value		
	0 d	21 d	35 d		0 d	21 d	35 d		PSO (P)	Days (D)	P × D
Milk fat	5.2	5.0	5.3	0.13	5.6	4.2	4.2	0.20	0.021	< 0.01	0.013
4% FCM (kg/day)	38.0	35.4	37.5	1.89	44.4	38.5	38.0	2.32	0.291	0.487	0.734
Milk fat (kg/day)	1.7	1.5	1.7	0.09	2.0	1.6	1.5	0.11	0.576	0.205	0.450
Milk protein	3.4	3.2	3.3	0.04	3.4	3.2	3.4	0.04	0.572	0.007	0.719
Lactose	4.7	4.4	4.7	0.03	4.7	4.4	4.8	0.04	0.973	0.022	0.732
SNF	8.6	8.3	8.7	0.05	8.7	8.2	8.8	0.07	0.694	0.015	0.626
SCs	106.7	193.3	208.5	41.94	128.4	628.2	233.5	141.12	0.277	0.259	0.421
MUN	18.6	17.0	20.7	0.36	18.1	12.4	16.8	0.47	< 0.01	< 0.01	< 0.01
Acetone	0.003	0	0.007	0.00	0	0	0.026	0.01	0.146	< 0.01	0.062
BHB	0.001	0	0	0.00	0	0	0.003	0.00	0.531	0.502	0.283
Β-Casein	2.6	2.5	2.6	0.03	2.67	2.5	2.62	0.04	0.764	0.011	0.618

^1^PSO: group fed TMR with 0.1% PO and 2% SBO as DM basis.

^2^Items: SNF, solid-not-fat; MUN, milk urea nitrogen; BHB, B-Hydroxybutyrate.

**Table 6:** Effects of feeding mixed phytoncide essential oil and soybean oil to dairy cows for 35 days on milk fatty acid compositions.

items		Control		SEM		PSO^1^		SEM	P-value		
	0 d	21 d	35 d		0 d	21 d	35 d		PSO (P)	Days (D)	P × D
% of total FAs											
STA (stearic acid)	0.12	0.19	0.12	0.010	0.14	0.15	0.17	0.010	0.049	0.017	0.295
TVA (trans vaccenic acid)	3.03	1.39	0.83	0.417	2.24	1.20	1.10	0.589	< 0.01	< 0.01	< 0.01
C18:2n6t (linolelaidic)	0.45	0.81	0.21	0.051	0.42	0.80	0.37	0.072	< 0.01	< 0.01	0.003
C18:2n6c (linoleic)	0.18	0.15	0.03	0.181	0.42	0.09	0.04	0.162	0.146	0.004	0.378
cis-9, trans-11-CLA	0.29	0.38	0.37	0.031	0.39	0.55	0.65	0.044	0.437	< 0.01	< 0.01
trans-10, cis-12-CLA	0.02	0.02	0.01	0.011	0.06	0.02	0.05	0.015	0.048	0.311	0.041
SFA (saturated fatty acids)	36.26	37.94	31.54	1.124	33.66	32.67	25.42	1.215	< 0.01	< 0.01	0.547
UFA (unsaturated fatty acids)	63.57	61.41	68.28	1.140	68.11	67.90	74.34	1.634	< 0.01	0.014	0.911
PUFA	1.64	2.08	1.42	0.103	2.29	2.39	2.10	0.076	< 0.01	< 0.01	0.387
CLA	0.31	0.39	0.38	0.035	0.45	0.57	0.70	0.049	0.922	< 0.01	< 0.01

^1^PSO = group fed TMR with 0.1% PO and 2% SBO as DM basis.

*CLA* conjugated linoleic acid.

#### CH_4_ emission analysis

**Figure 2:**
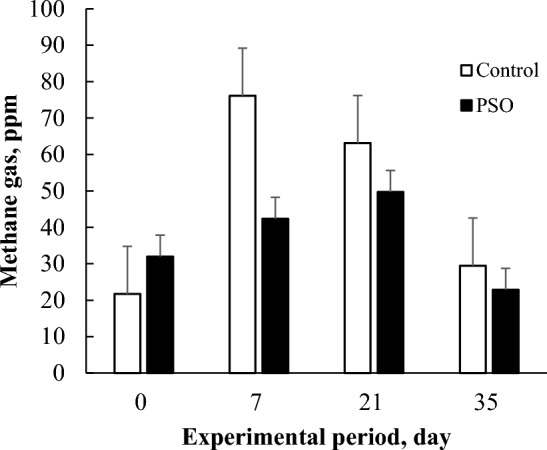
Effects of feeding mixed phytoncide essential oil and soybean oil to dairy cows for 35 days on methane gas (CH_4_). Values are expressed as the mean of methane production from all cows in each treatment.

The analysis of CH_4_ emission from the cows was conducted by using laser detector in each group (Fig. 2). There were 4 detection times during the experiment at (0, 7, 21, 35) days. Although the data could not be analyzed statistically because we measured in total for each group with 3 replicates, we could find that lower CH_4_ gas emissions was observed in the treatment group than in the control group from 7 to 35 days.

## Discussion

### In vitro experiment

This in vitro experiment was conducted to select optimal dosage of PO and SBO which could be used in in vivo experiment by considering the effect of the oils on production of CH_4_, CLA and influence on ruminal fermentation.

In this experiment, ruminal pH was not affected by PO or SBO (Table 1). Some studies have reported that ruminal pH could be variously changed by high-concentrate diets in feed and the addition of fat source. High-concentrate diets supplemented with sunflower oil can promote the fermentation of rumen microbes and consequently decrease ruminal pH^32^. Doreau and Ferlay^33^ have reported that ruminal pH does not change even in high-energy diets such as SBO. As mentioned before, the variety of ruminal pH could be affected by changes of ruminal fermentation patterns. Considering that neither SBO nor PO affected ruminal pH value, these sources might not directly affect the environment of ruminal fermentation.

Results of our experiment showed that there was no significant difference in the amount of ammonia-N concentrate in the group treated with 0% PO at 48 h (Table 1). However, ammonia-N was affected by SBO (increase of ammonia in SBO supplementation group with PO at 0.1%). The synthesis of ruminal ammonia nitrogen is also known to be influenced by the composition of fat source. Beauchemin et al.^34^ reported that the supplemented fat sources can be used as an energy booster for rumen microbes, affecting ruminal fermentation patterns and the synthesis of microbial protein. However, it is also known that fat additives, which can be used as an energy booster, have various effects on ruminal pH and microbial activity, depending on the fat composition^33^. In this study, when considering that the ammonia-N concentrate was numerically higher in 0% than in 0.1% PO treatments and the ammonia-N was increased by the SBO at 0.1% PO treatments, it is speculated that PO and SBO affected the synthesis of microbial protein and ammonia-N differently.

Generally, total gas production and CH_4_ levels in rumen are negatively correlated in in vitro experiments with fat supplements. This is because the fat source can facilitate ruminal lipolysis, resulting in decreased CH_4_ and increased total gas production by affecting ruminal microbes^12,23^. Wettstein et al.^35^ have reported that fat supplement in diet can reduce total gas production, which means exhausted energy. Thus, they suggested that fat supplements could improve the energy availability of microorganisms by reducing the exhausted energy in rumen. On the other hand, it has been reported that the addition of unsaturated fatty acids in feeds can act as a hydrogen sink in the rumen besides lipolysis, inducing hydrogen ions that could be used for CH_4_ production, to the process of converting unsaturated fatty acid into saturated fatty acid^23^.

In addition, as with PO, the essential oil has antimicrobial effects in the rumen, and results in lowering gas production during ruminal fermentation^36^. Considering all these phenomena, the results of this study indicate that the reduced total gas and CH_4_ production were affected by the hydrogen sink effect and especially accelerated the lipolysis effect of the oil; the effect could be confirmed more severely at 0.1% PO and 2% SBO addition (Table 1).

Factors affecting VFA compositions in association with the rumen fermentation environment are various, including the ratio of forage to concentrate, high protein, carbohydrate and fat content in feed, supplemented fat composition, and so on^35,37^. Depending on the fermentation rate of feed in rumen, ruminal pH and VFA composition changes are associated with each other. For example, the rate of fermentation in the rumen in the case of overfeeding concentrate could be faster than that in the case of feeding low concentrate, resulting in lower ruminal pH, lower levels of acetate and butyrate, and increased propionate ratio in the VFA composition. In particular, it has been reported that oil supplement in feed can be fermented by ruminal microbes and used as an energy booster, thereby modifying the VFA composition by reducing the pH in the rumen^38^. As previously mentioned, the addition of unsaturated fatty acids in the rumen can increase the production of propionate, act as a hydrogen sink, and reduce CH_4_ production. Castillejos et al.^39^ have reported that antimicrobial materials such as essential oil can increase the ratio of acetate to propionate in calves fed a 6:4 alfalfa hay:concentrate diet. In contrast, Cardozo et al.^40^ have observed that the ratio of acetate to propionate is decreased in calves fed a 1:9 straw: concentrate (based on corn, barley, and soybean meal) diet supplemented with essential oil. It has been reported that effects of essential oil on ruminal pH and feed composition are very diverse^36^. Considering these results, further research is needed to determine the effect of plant-derived essential oil on ruminal VFAs.

In results of long chain fatty acids in rumen, consecutive changes of linoleic acid were observed (Table 3). In the early stage of fermentation, TVA from linoleic acid was increased by the SBO supplement. CLA was increased by SBO and PO. Linoleic acid, which is found in SBO, is known to produce CLA and TVA through isomerization and biohydrogenation by microorganisms in the rumen^41^. According to Kairenius et al.^42^, fatty acid metabolism is affected by the composition of the added fat. A higher degree of unsaturation of the added fat causes more extensive metabolism of fatty acids. In addition, Kim et al.^16^ have reported that the addition of not only fat, but also antimicrobial essential oil such as PO, can induce an increase of CLA in the milk of ruminants. Taking all these studies into account, the increase of CLA in the present study might be due to effects of SBO containing a large amount of unsaturated fatty acids and PO. Furthermore, it was speculated that the most activation of inducing hydrogen sink might have occurred by 0.1% PO and 2% SBO addition when considering most decreases in CH_4_ and VFAs and increase in CLA production simultaneously.

### In vivo experiment

This in vivo experiment was carried to confirm the effect of selected supplementation (0.1% PO and 2% SBO) in in vitro experiment on increasing milk CLA and depressing CH_4_ emission by using dairy cows.

As expected from results of in vitro experiment (Table 6), CLA percentage and CLA yield were higher in the PSO group. According to Wang et al.^41^, CLA in milk could be increased when linoleic acids, the same as SBO, are added to the diet. The problem is that increased t10, c12 CLA often decreases milk fat synthesis. In the current study, milk fat percentage was decreased. However, milk yield was higher in PSO group than control thus, amount of milk fat (kg/day) was not different between two groups. In addition, total CLA yield was not decreased, although t10, c12 CLA was higher in the treatment group than that in the control group. Considering all these phenomena, PO might have induced more c9, t11 CLA isomerization than t10, c12 CLA isomerization by affecting related microbes (e.g., *Butyrivibrio fibrisolvens*). In addition, increased UFA and PUFA levels were observed at the same time in the treatment group. All these differences between the two groups could be explained by the amount of linoleic acid in added SBO and PO inducing hydrogen ion to isomerization of linoleic acid from what might be methanogenesis results of in vitro experiment. Thus, induced isomerization in the treatment group might have increased UFA, PUFA, and CLA compared to the control group. In addition, stearic acid was higher in the PSO group at 35 days. This higher stearic acid could be explained by greater microbe biohydrogenation of linoleic acids in the PSO group fed more linoleic acid than the control group.

The limitation of this study was measuring CH_4_ per pen but not in individual animals. However, to cover this limitation we measured the CH_4_ 3 times during the experiment. Interestingly, we observed the adaptability of cows to the PSO treatment, as it showed considerable increase in 7 days, and gradual increase after 7 days until the end of experiment (Fig. 2). Further analysis with individual measurement of methane production may be performed to warrant the obtained result. Generally, the production of CH_4_ in ruminant is decreased when dietary fat is added, and it is reported that the effect varies greatly, depending on the constituents or unsaturation of added fat^12,23^. As mentioned earlier, the addition of unsaturated fatty acids is known to induce fatty acid metabolism and inhibit CH_4_ production in rumen microbes. In the result of gas production in in vitro experiment, it is observed that the addition of SBO, which contains a large amount of unsaturated fatty acid, decreased CH_4_ production, and PO addition resulted in more decrease in CH_4_. Considering the results of fatty acid in rumen and milk, the CLA enhancement effect of the two oil sources confirmed in this study may ultimately lead to a decrease in CH_4_ production, by competition of hydrogen ion between biohydrogenation and CH_4_ production.

Complete blood cell counting (CBC) is a representative physiological index of animals. It was used in this study for the purpose of verifying the safety of experimental additives through animals. Some researchers have shown that oil supplements can affect animal disease because of the negative effect of unsaturated fatty acid in rumen^43^. However, in this study, there were no significant differences in results of CBC (white blood cell, lymphocyte, monocyte, or other indices) between control and treatment groups containing PO and SBO. Thus, the addition of PO and SBO supplement to the diet did not lead to disease-related chemical changes in dairy cows (Supplemental Table S2).

Some researchers have found a relationship between fat supplement and blood metabolites^44,45^. According to Henderson et al.^43^, fat addition in ruminant diet affects the synthesis of microbial protein in rumen. The added fat is used as an energy booster for protein synthesis, resulting in increasing amino acid-N and decreasing ammonia-N in the rumen, eventually reducing nitrogen content in the blood. It is known that fat degraded by lipolysis in rumen is fermented to increase propionate content in VFA compositions, thereby increasing glucose content in the blood by gluconeogenesis^46^. Similar to results of previous studies, the present study also showed that the PSO group containing oil supplement in feed had lower blood BUN but higher glucose concentration than the control group (Supplemental Table S3). Blood magnesium was also higher in the oil supplement group compared to the control. In general, most of the digestive and absorption lipid is transferred itself or binding to protein. However, some proportions of the lipid binding to magnesium are presented in blood^47^. Thus, the higher blood magnesium in our result might be affected by the oil supplement in diet^47^. The reason why blood Ca showed a tendency to be significant was unknown. This parameter was inconsistent with other plasma outcomes.

Fecal microbes are microorganisms involved in the digestive physiology of livestock. Their digestibility have been evaluated according to their populations^48^. Currently, antimicrobial effects of the essential oil PO have been extensively studied in rumen because it contains secondary metabolites, terpenoid, phenols, tannins, and alkaloids components which have antibacterial effects^49^. However, their effects on post-ruminal digestive have not been studied in detail yet. Furthermore, although the essential oil PO used in the experiment is also known to have antimicrobial effects, it is not yet known whether such effect could affect the whole ruminant digestive system^50^. However, as shown in the present study, numbers of pathogens in fecal microbes decreased due to the addition of PO and SBO. This demonstrates that the antimicrobial effect of PO also affects post-ruminal digestive system. Thus, the result of decrease in pathogenic microbes by PSO could support that PO might also be able to improve the energy availability of feed in ruminant (Fig. 1).

Generally, some studies have reported that milk production is positively correlated with the energy content in feed^50^. As expected, this study also showed that the addition of 2% fat source, a high energy booster, resulted in an increase in milk yield. Bauman^50^ has reported that the increase in glucose in the blood is positively correlated with milk yield. In particular, the fat supplement used in this study increased glucose in the blood by increasing propionate in the VFA composition due to the effect of SBO and PO on rumen fermentation in in vitro experiment. Furthermore, the antimicrobial effect of PO might have affected fecal microbes. Thus, PO might be able to increase milk yield by improving the metabolizable energy of SBO and basal diet (Table 4).

From milk composition data (Table 5), milk fat content (kg/day) and 4% FCM were similar between control and treatment groups. This is different from the result of Rico and Harvatine^51^. This might be because SBO has an effect on milk fat depression by increasing trans-10, cis-12 CLA known to inhibit the synthesis of milk fat. In our result, the unchanged milk fat in the treatment group may be explained by the effect of PO on isomerization to cis-9, trans-11 CLA rather than to trans-10, cis-12 CLA. Milk nitrogen is generally influenced by blood nitrogen and ammonia-N in rumen. Thus, decreased MUN in the PSO group could be explained by the flow of decreased BUN in blood (Supplemental Table S3).

In summary, a mixture of 0.1% phytoncide oil (PO) and 2% soybean oil (SBO) in diet can lead to an increase in CLA and a decrease in CH_4_ production in rumen by inducing hydrogen ion from CH_4_ production to biohydrogenation, especially isomerization of linoleic acid with no change in milk fat. Therefore, a combination of PO and SBO could result in increased milk CLA and depressed CH_4_ production with no influence on milk fat.

### Supplementary Information

Supplementary Tables.

## Data Availability

Datasets generated during and/or analyzed during the current study are available from the corresponding author upon reasonable request.
