# Supplementary material for: Dietary supplementation of phytoncide and soybean oil increases milk conjugated linoleic acid and depresses methane emissions in Holstein dairy cows
Source: Sci Rep. 2024 Mar 5;14:5439. doi: 10.1038/s41598-024-53799-2 (PMC10914803; doi:10.1038/s41598-024-53799-2)
Supplement: Supplementary file 1 — Supplementary Tables. [file 41598_2024_53799_MOESM1_ESM.docx]

| **Supplemental Table S1**. Compositions of basal diet used for *in vivo* trial | | |
| --- | --- | --- |
| Basal diet | Control | PSO^1^ |
| Concentrate (kg) | 4.27 | 4.27 |
| Roughage (kg) | 2.42 | 2.42 |
| TMR (kg) | 22.20 | 22.20 |
| Vehicle (kg) | 2.70 | 2.70 |
| Soybean Oil (kg) | 0 | 0.57 |
| Phytoncide Oil (kg) | 0 | 0.029 |
| Chemical composition |  |  |
| DM, % | 77.38 | 77.36 |
| Crude protein, % | 14.50 | 14.47 |
| Ether extract, % | 3.78 | 5.76 |
| Crude fiber, % | 15.50 | 15.37 |
| Crude ash, % | 15.82 | 16.01 |
| NDF, % | 32.59 | 32.48 |
| ADF, % | 19.19 | 19.08 |
| Ca, % | 0.60 | 0.598 |
| P, % | 0.31 | 0.30 |
| NE_l_^2^, Mcal/d | 40.90 | 41.10 |
| ^*^DM: Dry matter, NDF: Neutral detergent fiber, ADF: Acid detergent fiber.  ^1^PSO = group fed TMR with 0.1 % PO and 2% SBO as DM basis | | |

^2^NE_l_, Mcal/d was estimated based on NRC (2001).

| **Supplemental Table S2**. Hematological analysis of blood fraction drawn from the jugular vein of daily cow given diet containing 0.1% phytoncide and 2% soybean oil | | | | | | | | | | | | |
| --- | --- | --- | --- | --- | --- | --- | --- | --- | --- | --- | --- | --- |
| Items^1^ |  | Control | | | SEM | PSO^2^ | | | SEM | P-value | | |
|  |  | 0 d | 21 d | 35 d |  | 0 d | 21 d | 35 d |  | PSO  (P) | Days  (D) | P x D |
| WBC (4-12) K/uL |  | 12.99 | 11.04 | 12.86 | 0.785 | 12.19 | 9.99 | 11.25 | 0.591 | 0.248 | 0.216 | 0.942 |
| Lymphocyte (2.5–7.5) K/uL |  | 8.34 | 8.52 | 10.52 | 0.8 | 7.78 | 6.93 | 8.72 | 0.681 | 0.222 | 0.308 | 0.878 |
| Monocyte (0–0.84) K/uL |  | 0.99 | 0.44 | 0.34 | 0.109 | 0.78 | 0.47 | 0.52 | 0.098 | 0.989 | 0.014 | 0.514 |
| Granulocyte (0.6–6.7) K/uL |  | 3.86 | 2.08 | 2.00 | 0.255 | 3.63 | 2.58 | 2.00 | 0.299 | 0.796 | <0.001 | 0.686 |
| RBC (5–10) M/uL |  | 7.01 | 6.96 | 7.15 | 0.111 | 7.34 | 6.97 | 7.15 | 0.14 | 0.542 | 0.605 | 0.706 |
| Hemoglobin (8–15) g/dL |  | 11.79 | 12.02 | 12.82 | 0.207 | 11.78 | 11.71 | 12.21 | 0.21 | 0.291 | 0.089 | 0.703 |
| Hematocrit (24–46) % |  | 33.90 | 32.43 | 34.91 | 0.568 | 33.58 | 32.04 | 32.45 | 0.586 | 0.198 | 0.237 | 0.476 |
| MCV (40–60) fL |  | 48.90 | 46.70 | 48.70 | 0.854 | 46.11 | 46.20 | 45.50 | 0.725 | 0.064 | 0.752 | 0.585 |
| MCH (11–17) pg |  | 16.93 | 17.33 | 17.94 | 0.271 | 16.12 | 16.84 | 17.13 | 0.242 | 0.055 | 0.079 | 0.915 |
| MCHC (30–36) g/dL |  | 34.81 | 37.16 | 36.71 | 0.28 | 35.00 | 36.55 | 37.79 | 0.402 | 0.598 | <0.001 | 0.259 |
| Platelet (100–800) K/ul |  | 424.20 | 344.70 | 385.20 | 24.82 | 389.70 | 352.60 | 433.00 | 25.219 | 0.843 | 0.298 | 0.642 |
| ^1^Item: WBC, white blood cell; RBC, red blood cell; MCV, mean corpuscular volume; MCH, mean corpuscular hemoglobin; MCHC, mean corpuscular hemoglobin concentration.  Values are expressed as mean ± standard error of the mean.  ^2^PSO: group fed TMR with 0.1% PO and 2% SBO as DM basis  ^3^Normal range. | | | | | | | | | | | | |

| **Supplemental Table S3**. Changes in metabolic parameters of blood in dairy cows given diet containing a mixture of 0.1% PO and 2% SO for 35 days | | | | | | | | | | | | |
| --- | --- | --- | --- | --- | --- | --- | --- | --- | --- | --- | --- | --- |
| MPT^2^ | Control | | | SEM | PSO^1^ | | | SEM | P-value | | | |
|  | 0 d | 21 d | 35 d |  | 0 d | 21 d | 35 d |  | PSO  (P) | Days  (D) | P x D |  |
| ALB | 3.8 | 4.9 | 4.4 | 0.37 | 4.5 | 4.2 | 3.9 | 0.52 | 0.146 | 0.974 | 0.042 |  |
| GOT | 88.4 | 116.1 | 99.5 | 12.28 | 94.0 | 97.9 | 97.3 | 17.37 | 0.958 | 0.482 | 0.703 |  |
| GPT | 33.2 | 44.1 | 36.1 | 3.30 | 34.7 | 32.7 | 32.7 | 4.67 | 0.872 | 0.87 | 0.374 |  |
| BUN | 26.0 | 20.1 | 19.8 | 1.06 | 23.1 | 18.0 | 18.6 | 1.49 | 0.029 | <0.01 | 0.335 |  |
| CREA | 0.8 | 0.9 | 0.8 | 0.03 | 0.9 | 0.9 | 0.9 | 0.04 | 0.581 | 0.741 | 0.683 |  |
| TG | 8.4 | 8.9 | 10.5 | 1.24 | 8.9 | 9.7 | 11.1 | 1.75 | 0.75 | 0.04 | 0.961 |  |
| CHO | 300.7 | 302.6 | 316.4 | 17.81 | 292.5 | 324.8 | 361.1 | 25.18 | 0.428 | <0.01 | 0.078 |  |
| GLU | 58.0 | 65.0 | 70.4 | 2.13 | 63.4 | 65.7 | 72.5 | 3.02 | 0.089 | <0.01 | 0.354 |  |
| CA | 9.6 | 8.8 | 9.2 | 0.18 | 9.1 | 9.0 | 9.2 | 0.25 | 0.095 | 0.247 | 0.091 |  |
| MG | 3.2 | 3.0 | 2.6 | 0.12 | 2.9 | 2.6 | 2.6 | 0.16 | 0.01 | <0.01 | 0.151 |  |
| NEFA | 155.8 | 276.8 | 289.6 | 43.19 | 155.9 | 173.4 | 180.4 | 61.08 | 0.856 | 0.031 | 0.132 |  |
| ^1^PSO: group fed TMR with 0.1% PO and 2% SBO as DM basis  ^2^MPT: ALB, albumin; GOT, Glutamic-oxaloacetic transaminase; GPT, Glutamic pyruvic Transaminase; BUN, Blood urea nitrogen; CREA, Creatinine; TG, Triglycerides; CHO, cholesterol; GLU, Glucose; CA, calcium; MG, magnesium; NEFA, Non ester fatty acid. | | | | | | | | | | | | |
